# Relationship Between Vitamin D Receptor Gene BsmI Polymorphism and 25-Hydroxyvitamin D Total Levels in Slovak Postmenopausal Women with Reduced Bone Mineral Density

**DOI:** 10.3390/genes16030337

**Published:** 2025-03-13

**Authors:** Marta Mydlárová Blaščáková, Zuzana Lőrinczová, Lenka Anderková, Olga Czerwińska-Ledwig, Ľudmila Mikulová, Hedviga Hrušovská, Bernadeta Jędrzejkiewicz, Anna Piotrowska

**Affiliations:** 1 Department of Biology, Faculty of Humanities and Naturel Sciences, University of Prešov in Prešov, Ul. 17 novembra 1, 080 01 Prešov, Slovakia; 2 Osteocentre, AGEL Hospital Košice-Šaca a.s., Lúčna 57, 040 15 Košice-Šaca, Slovakia; zuzana.lorinczova@nke.agel.sk; 3 Institute for Basic Sciences, Faculty of Physiotherapy, University of Physical Education, 31-571 Krakow, Poland; olga.czerwinska@awf.krakow.pl; 4 Department of Medical and Technical Disciplines in Health Care, Faculty of Health Care, University of Prešov in Prešov, Partizánska1, 080 01 Prešov, Slovakia; ludmila.mikulova@unipo.sk; 5 Department of Nursing, Faculty of Health Sciences, Vincent Pol University in Lublin, 20-001 Lublin, Poland

**Keywords:** 25(OH)D total, biomarkers, bone mineral density, gene variant BsmI, *VDR* gene

## Abstract

**Objectives:** The BsmI polymorphism of the *VDR* gene (vitamin D receptor) is one of the important genetic variants influencing the development of osteoporosis. Measurement and evaluation of the 25-hydroxyvitamin D (25(OH)D) concentration in individuals with reduced bone mineral density are essential because deficiency of this hormone causes impaired bone mineralization, leads to low BMD (bone mineral density), and influences fracture formation. The aim of the study was to investigate the relationship between the *VDR* gene BsmI polymorphism and 25(OH)D levels in Slovak postmenopausal women. **Materials and Methods:** The study population consisted of 287 untreated postmenopausal women, who were divided into three groups based on T-scores: normal (CG = 65), osteopenia (OPE = 126), and osteoporosis (OPO = 96). DNA isolation was performed using a standard protocol. Genetic analyses of the BsmI (rs1544410) polymorphism of the *VDR* gene were performed using the TaqMan SNP genotyping assays. Biochemical analysis of total 25(OH)D was performed in blood serum using the electrochemiluminescence method. **Results:** The chi-square test confirmed that the mutant T allele was not associated with the development of osteoporosis (*p* = 0.419). Through Kruskal–Wallis analysis, we found significant differences (*p* < 0.05, *p* < 0.01) in total 25(OH)D concentrations in individual genotypes of the BsmI variant of the *VDR* gene between the groups of women studied. **Conclusions:** It can be concluded that the *VDR* gene and its variant BsmI as well as 25(OH)D total may be relevant markers in the etiology of the search for individuals at risk of osteoporosis.

## 1. Introduction

Vitamin D is a fat-soluble pleiotropic hormone. It plays an important role in calcium homeostasis and bone metabolism and can modulate the function of innate and adaptive immunity [1,2]. The sources of vitamin D, including D2 and D3, include skin production through exposure to sunlight and dietary intake. Subsequently, vitamin D is converted to 25-hydroxyvitamin D [25(OH)D] by D-25-hydroxylase in the liver. 25(OH)D is further converted to its active form 1,25-dihydroxyvitamin D [1,25(OH)2D] in the kidney by the enzyme 25-hydroxyvitamin D 1α-hydroxylase [3,4]. Vitamin D regulates the activity of bone cells—osteoblasts and osteoclasts—and prevents excessive parathyroid hormone (PTH) secretion, thereby promoting bone tissue formation [5]. Disruption of this regulation results in poor osteoid mineralization [6]. Vitamin D deficiency in the body causes decreased absorption of calcium and phosphorus in the small intestine. Serum vitamin D shortage is compensated for by Ca mobilization from skeletal stores. This can lead to impaired bone mineralization [7].

Vitamin D deficiency is particularly closely associated with bone diseases such as osteoporosis and rachitis. Serum 25(OH)D measurement is currently considered the most reliable marker for monitoring vitamin D status in the body due to its long half-life in circulation [8]. Insufficiency (21–29 ng/mL) and deficiency (<20 ng/mL) of 25(OH)D are widespread worldwide, especially in postmenopausal women. The majority of postmenopausal women with osteoporosis suffer from bone loss associated with estrogen deprivation. Rapid bone loss results from an increased bone turnover, which is related to an imbalance between osteoformation and osteoresorption [9]. In addition, postmenopausal women are at high risk of osteoporosis and subsequent osteoporotic fractures due to vitamin D deficiency [10]. Osteoporosis and its associated complications are major global concerns, as the disease is a significant cause of morbidity and mortality and affects approximately 200 million women worldwide [11,12].

The *VDR* gene has a significant function in the modulation of vitamin D pathways and regulation of hormone-responsive genes [13]. It has been indicated that in addition to environmental factors, genetic factors may account for 23 to 80% of the variability in serum vitamin D levels, as observed in studies involving twins [14,15]. One of the genetic variants studied in the *VDR* gene and associated with vitamin D metabolite levels is the rs1544410 polymorphism, located in the intron region (intron 8 near the 3′ terminus). In addition to vitamin D levels, the BsmI polymorphism of the *VDR* gene has also been shown to be associated with obesity, insulin resistance, diabetes mellitus type 2 [16,17,18], increased risk of developing postmenopausal osteoporosis, and response to antiresorptive therapy in the populations studied [19]. Based on these facts, it is important to identify candidate gene variants that are responsible for low bone mineral density (BMD) and low vitamin D levels. These findings will then help design tailored clinical approaches aimed at preventing or at least delaying the development of osteoporosis. With this in mind, the present study aimed to investigate the association between the BsmI polymorphism (rs1544410) of the *VDR* gene and total 25(OH)D levels, as well as anthropometric and densitometric parameters in Slovak women with reduced bone mineral density.

## 2. Materials and Methods

### 2.1. Study Group

The research population consisted of 287 postmenopausal women without fractures from the Košice region (eastern Slovakia). The women involved in the study participated in a medical screening at the osteological outpatient clinic of AGEL Hospital, Košice-Šaca, a.s. To be included in the study, they had to meet the inclusion criteria: postmenopausal status, no vitamin D supplementation for at least six months before and during recruitment to the study, no history of menstrual disorders, and good physical and psychological condition. Women were excluded from the study when they met the exclusion criteria: premature menopause, genetic and metabolic bone diseases, rheumatoid arthritis, diabetes mellitus type I, BMI < 19 kg/m^2^, anorexia nervosa, primary hyperparathyroidism, corticosteroid therapy (treatment with prednisone 5 mg or more), chronic hepatopathy, chronic inflammatory bowel disease, chronic renal insufficiency, prolonged immobilization, Cushing’s syndrome, myeloproliferative disorders, etc.

Women who qualified for the project were divided into three groups based on densitometric measurements (DXA) and the WHO T-score classification (1994): results within the normal range (CG control group = 65 women), T-score ≥ 1 to −1; group with osteopenia (OPE = 126 women), T-score from −1 to −2.5; and group with osteoporosis (OPO = 96 women), T-score of −2.5 or more.

### 2.2. Densitometric Measurement—DXA

Densitometric screening was performed in all women of the study population in cooperation with the Osteocentrum, a.s., AGEL Hospital, Košice-Šaca, Slovakia. The densitometric screening was performed using a full-body DXA Hologic Discovery densitometer (Hologic Inc., Marlborough, MA, USA). The following densitometric parameters were measured:

- Total BMD, T-score, and Z-score in the left hip area;
- BMD, T-score, and Z-score in the left hip area;
- Total BMD, T-score, and Z-score in the lumbar spine (vertebrae L1-L4).

### 2.3. Biochemical Analysis

Peripheral blood samples were collected from April to June and from October to December 2024 and in January 2025. Blood samples were collected from participants on a fasting basis in S-Monovette^®^ collection tubes containing a clotting activator (SARSTEDT, Nümbrecht, Germany) at a volume of 5.5 mL. Blood samples were prepared by centrifugation at 4000 rpm/10 min using a Hettich MIKRO 200R centrifuge (Hettich, Kirchlengern, Germany). Total serum 25(OH)D and PTH concentrations were determined using kits (Elecsys^®^ Vitamin D total III, Elecsys^®^ PTH, Mannheim, Germany) via a Cobas e411 immunochemical analyzer (Roche, Tokyo, Japan). Inter-Assay CV Vitamin D total III: High % CV 18.48; Low % CV 10.33. Intra-Assay CV Vitamin D total III: % CV 14.41. Inter-Assay CV PTH: High % CV of Means 7.23; Low % CV of Means 2.24. Intra-Assay CV PTH: % CV 4.98.

### 2.4. Molecular Genetic Analysis

For molecular genetic analysis, a venous blood sample was collected in S-Monovette^®^ collection tubes containing an anticoagulant agent (SARSTEDT, Nümbrecht, Germany) with a volume of 2.7 mL, and DNA was isolated in the collected blood samples using the NucleoSpin^®^ Blood isolation kit (Machery-Nagel GmbH & Co. KG, Düren, Germany). Isolation was performed according to the protocol included with the commercial kit. Prior to genotyping, the concentration and purity of DNA were determined. DNA concentration was measured using a NanoDropTM 2000 spectrophotometer (Thermo Fisher, Wilmington, DE, USA). Genotyping analysis of the BsmI (rs1544410) polymorphism of the *VDR* gene was performed by real-time PCR using a 7500 Fast Real-Time PCR System analyzer (Applied Biosystems^®^, Foster City, CA, USA) via a standard protocol using a commercially produced TaqMan genotyping SNP Assay probe (C8716062_20) (Applied Biosystems^®^, Foster City, CA, USA). The risk genotype was verified.

### 2.5. Anthropometric Measurement

Measurement of body height was performed using a Seca 206 (Seca, Hamburg, Germany) personal altimeter with a rolling mechanism. Measurement of body weight was performed using a Seca 813 digital personal scale (Seca, Germany). Based on the measured values of body height (cm) and body weight (kg), the body mass index (BMI) was calculated.

### 2.6. Statistical Analysis

The measured values of anthropometric, densitometric, biochemical, and molecular genetic parameters were processed using Microsoft Excel 2011 (Microsoft, Redmond, WA, USA) and Statistica ver. 12 (Statsoft, Tulsa, OK, USA). Individual parameters were evaluated by means of statistical characteristics: position (mean) and variability (standard deviation). Significant differences between groups of women in the study population were detected using analysis of variance and Student’s *t*-test. Significant differences between groups of women were detected by parametric or non-parametric ANOVA based on the significance of the data. Correlations between total 25(OH)D and selected parameters were evaluated using Spearman correlation analysis. Chi-square, statistical significance, Hardy–Weinberg equilibrium, odds ratio (OR), and confidence interval (95% CI) values were obtained using the following online calculators: MedCalc (MEDCALC^®^ ver. 16.2.1), Hardy-Weinberg Equilibrium Calculator (https://www.sebc.me/bioblog/labs/hwe-calculator; accessed on 3 January 2025), and Social Science Statistics (www.socscistatistics.com; accessed on 23 November 2024). A *p*-value < 0.05 was set as significantly significant. Using the online OpenEpi calculator (https://www.openepi.com/SampleSize/SSPropor.htm, accessed on 2 March 2025), sample sizes were determined for 95% and 99.99% confidence levels for n = 287, assuming *p* = 50% ±5, (d) of 5%, and a DEFF of 1: CI 95%, n = 165 subjects; CI 99.99%, n = 242 subjects. The sample sizes obtained allowed the differences to be statistically valid.

## 3. Results

### 3.1. Results of Anthropometric Measurements and Biochemical Analyses

Table 1 shows the statistical evaluation of anthropometric and biochemical parameters. Significant differences between all groups of women in the parameters of age and body weight were revealed. In the parameter age, there were significant differences between OPO and CG (*p <* 0.001), OPO and OPE, and CG and OPE (*p <* 0.05). In body weight, significant differences were found between OPO and CG, OPO and OPE (*p <* 0.001), and OPE and CG (*p <* 0.05), while for BMI, significant differences were observed between OPO and CG and OPO and OPE (*p <* 0.001). The group of women with osteoporosis showed the lowest mean values of body weight and BMI. Based on the mean BMI value (28.89 ± 5.30 kg/m^2^), according to the WHO (1990), women in the study population could be classified as overweight. Women in the OPO and OPE groups were classified as overweight, while women in the CG group were classified as having class I obesity.

Average total 25(OH)D serum levels were lowest in the group of women with osteoporosis (17.04 ± 12.01 ng/mL). Based on the mean values of 25(OH)D total, the OPO and CG groups can be classified as deficient (10–20 ng/mL). The average values in the OPE group were within the range of values for deficiency status for the Slovak population (21–29 ng/mL).

The mean PTH level in the whole study population was 25.89 ± 15.37 pg/mL, which falls within the reference range of PTH levels. The lowest mean PTH value was noted in the group with osteopenia (20.41 ± 13.23 pg/mL), which is also in the reference range of PTH levels (14–72 pg/mL). The highest mean PTH level was in the OPO group, which also had the lowest mean level of total 25(OH)D.

### 3.2. Results of Densitometric Measurements

The monitored densitometric parameters of the left hip and lumbar spine were significantly different in the studied groups (*p* < 0.001) (Table 2). The mean values of all DXA parameters (T-score, Z-score, BMD) in the hip and lumbar spine were significantly lower in the group of women with osteoporosis compared to the mean values in the OPE and CG groups. All monitored densitometric parameters showed statistically significant differences between the three selected groups.

### 3.3. Results of Molecular Genetic Analysis

Table 3 shows the representation of the genotypes and alleles of the BsmI polymorphism of the *VDR* gene in the studied groups of women.

Based on the above genotype frequencies, it can be concluded that the TC genotype was observed with the highest frequency in all groups of women (OPO = 44.79%; OPE = 45.24%; CG = 47.69%). The TT genotype mutation was represented with the lowest frequency. Statistical analysis did not show a statistically significant difference in the genotype frequencies between the selected groups (*p* = 0.804).

When we monitored the allele frequency, we found that the C allele was represented with the highest frequency in the CG group (63.85%) and the lowest frequency in the OPE group (58.33%).

The T mutant was represented with the lowest frequency in the CG group (36.15%). Based on the statistical analysis, it was found that the T allele was not significantly associated with the incidence of osteoporosis in the studied population of women (*p* = 0.419; OR = 1.182; CI = 0.788–1.772).

Statistical analysis in the group of women with osteoporosis (OPO) and in the control group of women (CG) did not confirm significant differences between the genotypes of the *VDR* gene BsmI polymorphism in relation to the anthropometric, densitometric, or biochemical parameters studied (Tables S1 and S2).

In the OPE group, statistically significant differences were found between the genotypes of the *VDR* gene BsmI polymorphism in the spine Z-score parameter (*p* = 0.034) (Table 4). Through multiple comparisons of *p*-values, significant differences were also found between the TT and TC genotypes in the spine Z-score parameter (*p* < 0.05).

### 3.4. Effect of the VDR Gene BsmI Polymorphism on Total Vitamin D Levels

Table 4 and Tables S1 and S2 show the mean values of total 25(OH)D concentrations in individual genotypes of the BsmI variant of the *VDR* gene in the studied groups of women. The lowest average values of total 25(OH)D in the OPO and OPE groups were observed in the mutant TT genotype. However, statistical analysis did not confirm the existence of significant differences in total 25(OH)D between the different genotypes of the BsmI polymorphism of the *VDR* gene (GG: *p* = 0.164; OPE: *p* = 0.939; OPO: *p* = 0.809).

Based on the results of the non-parametric Kruskal–Wallis ANOVA and subsequent post-hoc tests, we confirmed statistically significant differences in the parameter of total 25(OH)D between the following groups: OPO genotype TT vs. OPE genotypes TC and CC (*p* < 0.01); OPO genotype TT vs. CG genotype CC (*p* < 0.05); OPO genotype TC vs. OPE genotypes TT (*p* < 0.05), TC, and CC (*p* < 0.01); OPO genotype TC vs. CG genotype CC (*p* < 0.05); OPO genotype CC vs. OPE genotypes TC and CC (*p* < 0.05); OPE genotype TC vs. CG genotype TC (*p* < 0.05); OPE genotype CC vs. CG genotype TC (*p* < 0.05).

Based on total 25(OH)D levels, the study population was divided into four categories: optimal vitamin D status, >30 ng/mL (n = 41); vitamin D insufficiency, 20–30 ng/mL (n = 92); vitamin D deficiency, 10–20 ng/mL (n = 99); and severe vitamin D deficiency, <10 ng/mL (n = 55). No statistically significant difference was confirmed between blood vitamin D levels according to the genotypes of the *VDR* gene BsmI polymorphism in the entire study population (χ^2^ = 12.241; df = 6; *p <* 0.057), nor in the individual OPO (χ^2^ = 2.132; df = 6; *p <* 0.907), OPE (χ^2^ = 8.729; df = 6; *p <* 0.189), and CG (χ^2^ = 8.451; df = 6; *p <* 0.207) study groups (Table 5).

Table 6 shows the distribution of the different groups of women based on the CC genotype and genotypes with at least one mutant (minor) TT + TC allele. In the next step, the effect of the TT + TC genotypes versus the CC genotype on total vitamin D levels in each group of women was examined. No relationship was confirmed between total vitamin D levels and the observed genotypes in the CG (χ^2^ = 5.582; *p <* 0.134), OPE (χ^2^ = 5.336; *p <* 0.149), and OPO (χ^2^ = 0.429; *p <* 0.934) groups, or in the total study population of women (χ^2^ = 5.964; *p <* 0.113) via chi-square test.

Based on the measured total 25(OH)D levels, there was a high prevalence of low vitamin D levels (<30 ng/mL) in the entire study population, up to 85.71%. Specifically, 32.06% of women had insufficiency, 34.49% had deficiency, and 19.16% had severe vitamin D deficiency. Only 14.29% of women had total vitamin D levels within a normal range.

### 3.5. Results of Correlation Analysis

In the CG and OPO groups, correlation analysis did not confirm the presence of a significant relationship between vitamin D concentration and selected anthropometric or densitometric parameters. In the OPE group, a significant relationship was found between vitamin D level and two densitometric parameters of the spine—T-score and BMD (Table 7). The value of the correlation coefficient confirms the existence of a weak, negative relationship between vitamin D concentration and both densitometric parameters of the spine, T-score (r = −0.1824; *p* = 0.042) and BMD (r = −0.1772; *p* = 0.048), in both cases.

**Figure 1:**
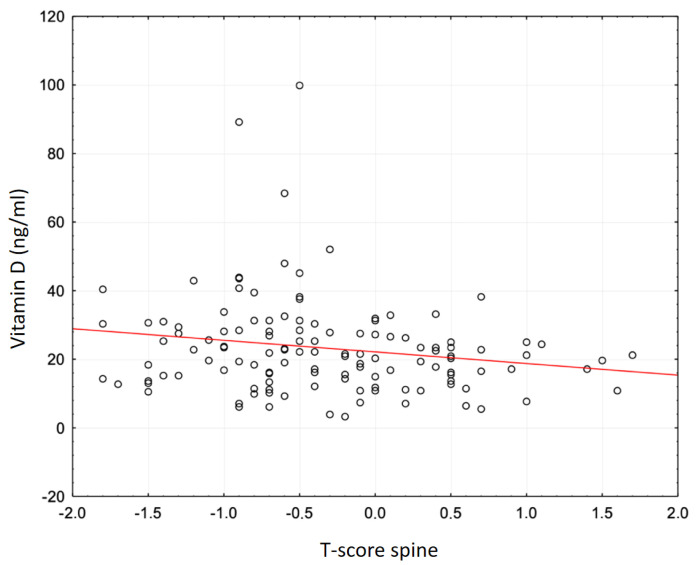
Correlation between 25(OH)D (vitamin D total) and total lumbar spine T-score in women with osteopenia.

**Figure 2:**
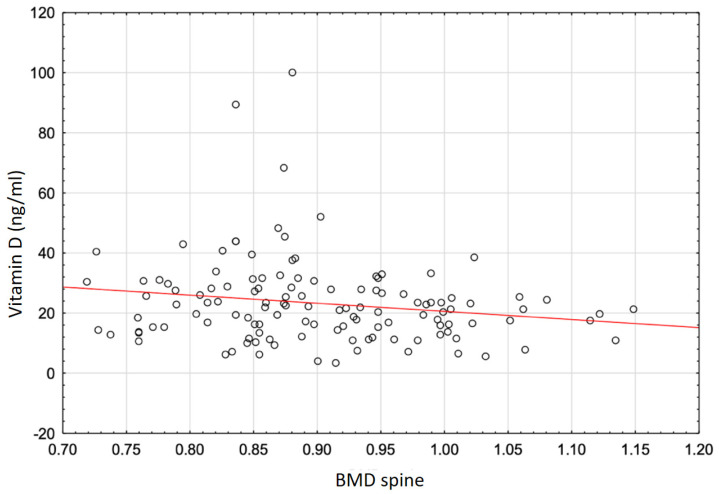
Correlation between 25(OH)D (vitamin D total) serum concentration and total lumbar spine BMD in women with osteopenia.

Figure 1 and Figure 2 present graphical representations of the correlations between serum total vitamin 25(OH)D concentration and total T-score and spine BMD.

## 4. Discussion

Osteoporosis is a multifactorial, polygenic disease that has a significant impact on disability and mortality worldwide. Due to the increasing life expectancy of the population and the increasing incidence of osteoporosis and related fractures, this disease is one of the most common metabolic bone diseases [20].

In addition to basic risk factors such as aging, low level of physical activity, poor lifestyle, smoking, premature menopause, positive family history, and low calcium intake and vitamin D [21,22,23], others factors like excessive body weight [24,25,26], body height loss [27], and high BMI [21,22,23,24,28,29] are important parameters that increase the risk of osteoporosis and osteoporotic fractures [30]. Several of these listed osteoporosis risk factors are also included in predictive models—FRAX [31], Garvan’s algorithm [32,33], and the Polish POL-RISK method [34]—which are useful in assessing the clinical condition of an individual and designing an effective therapeutic solution [27].

The prevalence of vitamin D deficiency in the Slovak Republic is high (over 50% of the population, increasing in winter). Therefore, it is important to assess its effect on BMD and use it as a prognostic marker of BMD loss. In this study, the effect of total vitamin 25(OH)D and PTH on bone mass in postmenopausal women was assessed. Significant differences in the studied groups were confirmed for both markers (Table 1). The mean value of total vitamin 25(OH)D concentration in the studied population was 20.24 ± 12.85 ng/mL, which is classified as vitamin D deficiency. The mean value of PTH in all probands was 25.89 ± 15.37 pg/mL, which does not indicate a PTH deficiency that could negatively affect vitamin D levels.

Based on the mean values of 25(OH)D total, it can be concluded that there is a high prevalence of vitamin D deficiency in all groups of women in the study population. The most pronounced vitamin D deficiency was in the OPO group (17.04 ± 12.01 ng/mL), which may indicate its negative effect on BMD. The associations of vitamin D total with BMD in women with osteoporosis were studied before [4,35,36]. Significant positive correlations between vitamin D levels and BMD and significantly negative correlations with PTH have been shown in the Saudi Arabian population [35]. In the Chinese female population, a high prevalence of vitamin D deficiency and insufficiency in women with osteoporosis or osteopenia was confirmed [4]. A negative association was also found between serum levels of the studied vitamin D form and bone turnover markers and PTH in a group of women with osteopenia and osteoporosis. In the OPO group, positive correlations were observed between vitamin 25(OH)D levels and femoral neck BMD (*p =* 0.010) and total hip BMD (*p =* 0.001). In the OPE group, higher vitamin 25(OH)D levels were associated with higher femoral neck BMD (*p =* 0.020) and total hip BMD (*p =* 0.008) and lower PTH (*p <* 0.001). In the study cohort, significant correlations were found between total vitamin 25(OH)D levels and densitometric markers only in the OPE group. These included a weak negative correlation between vitamin 25(OH)D total and spine T-score (r = −0.1824; *p <* 0.042) and an association of 25(OH)D total with spine BMD (r = −0.1772; *p <* 0.048) (Table 7, Figure 1 and Figure 2). Unlike the authors of the previous study, in the present study, no significant associations were observed between total vitamin 25(OH)D levels and femoral neck BMD or total hip BMD in any of the groups of studied women (*p* > 0.05). For spine BMD, no significant correlations were observed with total 25(OH)D levels in the OPO and CG groups. Differences in the results between previous studies and our own studies may be due to differences in ethnicity, age, and cut-off points for defining vitamin D deficiency and insufficiency, or undetected vitamin D supplementation.

The *VDR* gene is located on chromosome 12 (12q12-q14) and consists of nine coding exons [37]. The correlation of *VDR* gene polymorphisms with the development of civilization diseases, including osteoporosis, has been confirmed by several studies.

One of the most frequently studied polymorphisms of the *VDR* gene is the BsmI polymorphism (rs1544410), which, among other functions, may influence gene expression and differential biological responses to vitamin D [38,39,40]. In the present cohort of postmenopausal Slovak women, the T allele of the *VDR* gene BsmI polymorphism was not significantly associated with osteoporosis risk (χ^2^ = 1.262; *p =* 0.419) (Table 3). The heterozygous TC genotype occurred at the highest frequency in all groups of postmenopausal women studied (Table 3). There was no statistically significant difference in the distribution of genotypes between the study groups (χ^2^ = 1.624; *p =* 0.804). Similar results were also reported by some authors [41,42,43,44,45]. However, based on the results of other scientific studies [46,47,48,49,50], it can be concluded that the *VDR* gene and its variant rs1544410 may be a risk factor in the etiology of osteoporosis. This relationship is therefore still unexplained.

Several studies [40,51,52] have shown no relationship between the *VDR* gene BsmI polymorphism and fracture risk in postmenopausal women with osteoporosis. In postmenopausal women from southern Slovakia, it was found that the BsmI variant of the *VDR* gene significantly affects BMD of the lumbar spine [53]. Women with the genotype bb (here CC) variant of the *VDR* gene BsmI polymorphism had lower BMD values compared to heterozygous genotype Bb (TC) (*p <* 0.05). In the study group, there was a statistically significant difference between the genotypes of the variant of the *VDR* gene BsmI polymorphism and the studied parameters (anthropometric, densitometric, and biochemical) only in terms of the Z-score parameter, and this was in the OPE group (*p <* 0.032) (Table 4). The study of the influence of *VDR* gene polymorphisms on vitamin D status and BMD was also addressed in postmenopausal women from Belarus [54]. In the case of the rs1544410 variant of the *VDR* gene, the authors noted its significant effect on the concentration of total vitamin 25(OH)D in the blood. The dominant genotype of the *VDR* gene rs1544410 polymorphism in both groups of women was the TC genotype. An interesting finding was that the lowest 25(OH)D levels were in the original CC genotype, and the highest levels were found in probands of the mutant TT genotype. In the present study, the TC genotype also had the highest frequency (45.64%), and the least represented genotype in all the studied groups was the mutant TT genotype (OPO, 15.63%; OPE, 19.05%; CG, 12.31%) (Table 3). Divanoglou et al. [55] found in their study that individuals carrying the B allele (here T allele) of the BsmI polymorphism of the *VDR* gene (OR: 0.52; 95% CI: 0.27–0.99) had twice the risk of developing vitamin D deficiency compared to those with the b allele (here C allele).

Çakır et al. [56] found no significant differences (*p =* 0.602) between the genotypes of the BsmI variant of the *VDR* gene and the categories of vitamin 25(OH)D levels (optimal status, insufficiency, deficiency, and severe deficiency) in a cohort of Turkish postmenopausal women. This was confirmed by the present results of this survey of Slovak women. The least represented genotype in all the groups of studied women, divided on the basis of vitamin 25(OH)D levels, was the TT mutant genotype. The heterozygous TC genotype was present with the highest frequency in the severe deficiency (10.10%), deficiency (16.03%), and insufficiency (13.94%) groups. The most represented genotype in the insufficiency group was the reference genotype CC (15.33%) (Table 5). When each group of women was divided on the basis of genotypes, i.e., CC and genotypes with at least one mutant allele (TT + TC), no statistically significant association was confirmed between total vitamin D levels and genotypes in the groups of women studied (Table 6). Therefore, it can be concluded that the prevalence of low total vitamin 25(OH)D levels (<30 ng/mL) in the entire study cohort of postmenopausal women was as high as 85.71%. The observed differences between the present findings and those of other authors could be due to variations in ethnicity, distribution based on vitamin D metabolite levels, vitamin D intake (from sunlight or supplements), or other factors influencing total serum 25(OH)D vitamin or PTH levels in individuals. Further scientific studies should also focus on a more detailed analysis of dietary habits, sun exposure, and regular physical activity.

### Study Limitation

One of the major limitations of this study is that blood samples were taken in different seasons of the year. Nevertheless, this approach was required to collect such a large study group. Future studies may also take into account the seasons, bearing in mind that vitamin D deficiency may be more severe in autumn and winter. Other limitations of this study may include gene–gene and gene–environment interactions, epigenetic factors, and also the fact that the women involved in the scientific study were from only one area of the Slovak Republic.

## 5. Conclusions

The results of the present study show significant differences in total 25(OH)D concentrations in different genotypes of the BsmI variant of the *VDR* gene among the studied groups of postmenopausal women. There was a statistically significant relationship between total 25(OH)D concentration and densitometric parameters of the spine (T-score, BMD) in the group of women with OPE. Based on the molecular genetic analysis, the TC genotype was the most common in all groups of women, including healthy women and those with osteoporosis or osteopenia. The mutant T allele was not significantly associated with the occurrence of osteoporosis in the study population.

In addition, anthropometric parameters (weight, BMI), densitometric parameters (BMD, T-score), and biochemical markers (vitamin 25(OH)D total, PTH) may be useful markers in personalized medicine for screening postmenopausal osteoporosis and may serve as predictive biomarkers of osteoporosis.

## Figures and Tables

**Table 1:** Mean values of anthropometric and biochemical parameters in the studied groups of postmenopausal women.

	Total (n = 287)		OPO (n = 96)		OPE (n = 126)		CG (n = 65)		*p* Post-Hoc
	**Mean**	**SD**	**Mean**	**SD**	**Mean**	**SD**	**Mean**	**SD**	
Anthropometric Parameters									
Age (years)	62.83	9.42	65.98	9.20	62.60	8.80	58.63	9.31	0.001 * OPO vs. OPE: *p* = 0.019 OPO vs. CG: *p* < 0.001 OPE vs. CG: *p* = 0.013
Age at onset of menopause (years)	48.62	4.91	48.24	5.29	48.43	4.82	49.54	4.44	0.120
Body weight (kg)	74.27	13.17	67.71	10.73	75.23	11.15	82.09	15.26	0.001 * OPO vs. OPE: *p* < 0.001 OPO vs. CG: *p* < 0.001 OPE vs. CG: *p* = 0.010
Body height (cm)	160.51	6.65	159.66	7.06	160.28	6.25	162.19	6.58	0.053
BMI (kg/m^2^)	28.89	5.30	26.61	4.42	29.36	4.52	31.38	6.49	0.001 * OPO vs. OPE: *p* < 0.001 OPO vs. CG: *p* < 0.001
Biochemical Parameters									
PTH (pg/mL)	25.89	15.37	32.32	17.22	20.41	13.23	27.02	12.28	0.001 * OPO vs. OPE: *p* < 0.001 OPE vs. CG: *p* = 0.003
25(OH)D total (ng/mL)	20.24	12.85	17.04	12.01	23.23	14.16	19.20	9.91	0.001 * OPO vs. OPE: *p* < 0.001

Note: Total—all postmenopausal women; OPO—osteoporosis; OPE—osteopenia; CG—normal (control group); *p*—statistical significance *; SD—standard deviation; BMI—body mass index; PTH—parathyroid hormone; 25(OH)D total—vitamin D total.

**Table 2:** Mean values of densitometric parameters in the studied groups of postmenopausal women.

	Total (n = 287)		OPO (n = 96)		OPE (n = 126)		CG (n = 65)		*p*
	Mean	SD	Mean	SD	Mean	SD	Mean	SD	
Left hip–neck									
T-score	−1.29	1.09	−2.13	0.84	−1.32	0.69	0.01	0.73	0.001 *
Z-score	0.11	1.04	−0.57	0.85	0.05	0.71	1.24	0.88	0.001 *
BMD (g/cm^2^)	0.71	0.12	0.61	0.09	0.70	0.08	0.85	0.08	0.001 *
Left hip total									
T-score	−0.35	1.06	−1.14	0.87	−0.33	0.77	0.80	0.69	0.001 *
Z-score	0.78	1.03	0.18	0.94	0.74	0.81	1.74	0.81	0.001 *
BMD (g/cm^2^)	0.90	0.13	0.81	0.11	0.90	0.09	1.04	0.09	0.001 *
Spine total									
T-score	−1.06	1.48	−2.44	0.69	−0.88	1.02	0.63	1.14	0.001 *
Z-score	0.53	1.55	−0.67	0.95	0.69	1.28	2.01	1.36	0.001 *
BMD (g/cm^2^)	0.93	0.16	0.78	0.08	0.95	0.11	1.12	0.13	0.001 *

Note: Total–all postmenopausal women; OPO—group of women with osteoporosis; OPE—group of women with osteopenia; CG—control group; n—number; Mean—mean; SD—standard deviation; *p*—statistical significance *.

**Table 3:** Genotype and allele frequencies of the *VDR* gene BsmI polymorphism in the studied groups of women.

	Genotype							HWE		χ^2^	*p*
	TT		TC		CC						
	n	%	n	%	n		%				
OPO	15	15.63	43	44.79	38		39.58	0.627		1.624	0.804
OPE	24	19.05	57	45.24	45		35.71	0.436			
CG	8	12.31	31	47.69	26		40.00	0.790			
	**Allele**							**χ^2^**	***p***	**OR**	**CI**
	**T**			**C**							
	**n**	**%**		**n**		**%**					
OPO	73	38.02		119		61.98		1.262	0.419	1.182	0.788–1.772
OPE	105	41.67		147		58.33					
CG	47	36.15		83		63.85					

Note: OPO—group of women with osteoporosis; OPE—group of women with osteopenia; CG—control group; n—number; %—percentage; HWE—Hardy–Weinberg equilibrium; χ^2^—chi-square test value; *p*—statistical significance; OR—odds ratio; CI—confidence interval.

**Table 4:** Mean values of the monitored parameters in individual genotypes of the *VDR* gene BsmI polymorphism in the OPE group.

Parameter	Genotype						*p*
	TT		TC		CC		
	n = 24		n = 57		n = 45		
	Mean	SD	Mean	SD	Mean	SD	
Age (years)	63.92	5.23	62.02	9.73	62.64	12.25	0.386
Onset of menopause (years)	47.29	4.52	48.33	5.42	49.16	6.73	0.235
Body weight (kg)	76.98	11.01	74.76	11.99	74.89	12.76	0.727
Body height (cm)	158.85	6.15	160.89	6.30	160.27	22.22	0.409
BMI (kg/m^2^)	30.57	4.43	28.95	4.87	29.22	5.89	0.169
T-score LH neck	−1.30	0.73	−1.28	0.71	−1.39	0.72	0.738
T-score LH total	−0.27	0.91	−0.34	0.72	−0.35	0.77	0.921
Z-score LH neck	0.18	0.67	0.03	0.74	0.02	0.69	0.496
Z-score LH total	0.89	0.85	0.66	0.81	0.75	0.81	0.538
BMD LH neck	0.70	0.08	0.71	0.08	0.70	0.12	0.723
BMD LH total	0.91	0.11	0.90	0.09	0.90	0.09	0.943
T-score spine	−0.52	1.35	−1.01	1.00	−0.92	0.82	0.155
Z-score spine	1.20	1.54	0.47	1.31	0.68	0.98	0.034 * TT vs. TC: *p* = 0.034
BMD spine	0.99	0.15	0.94	0.11	0.94	0.09	0.159
PTH (pg/mL)	19.66	10.79	19.78	13.80	21.60	13.85	0.773
25(OH)D total (ng/mL)	22.75	11.97	23.39	15.47	23.27	13.80	0.939

Note: n—number; SD—standard deviation; BMI—body mass index; kg—kilograms; cm—centimeters; LH—left hip; PTH—parathyroid hormone; 25(OH)D total—vitamin D total; *p*—statistical significance *.

**Table 5:** Distribution of *VDR* gene BsmI polymorphism genotypes in the studied groups of women based on total vitamin D levels.

	Optimal Condition		Insufficiency		Deficiency		Significant Deficiency		χ^2^	*p*
Genotype	n	%	n	%	n	%	n	%		
Control Group (n = 65)										
TT	2	3.08	1	1.54	4	6.15	1	1.54	8.451	0.207
TC	2	3.08	9	13.85	12	18.46	8	12.31		
CC	5	7.69	11	16.92	4	6.15	6	9.23		
Osteopenia (n = 126)										
TT	8	6.35	3	2.38	11	8.73	2	1.59	8.729	0.189
TC	12	9.52	17	13.49	21	16.67	7	5.56		
CC	8	6.35	20	15.87	14	11.11	3	2.38		
Osteoporosis (n = 96)										
TT	0	0.00	4	4.17	7	7.29	4	4.17	2.132	0.907
TC	2	2.08	14	14.58	13	13.54	14	14.58		
CC	2	2.08	13	13.54	13	13.54	10	10.42		
Total (n = 287)										
TT	10	3.48	8	2.79	22	7.67	7	2.44	12.241	0.057
TC	16	5.57	40	13.94	46	16.03	29	10.10		
CC	15	5.23	44	15.33	31	10.80	19	6.62		

Note: n—total number; %—percentage representation; χ^2^—chi-square value; *p*—statistical significance; Total—total study population of postmenopausal women.

**Table 6:** Distribution of genotypes of the *VDR* gene BsmI polymorphism containing/not containing the mutated allele in the studied groups of women with respect to total vitamin D levels.

Category	Total n	Genotypes				χ^2^	*p*
		CC		TT + TC			
		n	%	n	%		
Control Group (n = 65)							
Optimal condition	9	5	55.56	4	44.44	5.582	0.134
Insufficiency	21	11	52.38	10	47.62		
Deficiency	20	4	20.00	16	80.00		
Significant deficiency	15	6	40.00	9	60.00		
Osteopenia (n = 126)							
Optimal condition	28	8	28.57	20	71.43	5.336	0.149
Insufficiency	40	20	50.00	20	50.00		
Deficiency	46	14	30.43	32	69.57		
Significant deficiency	12	3	25.00	9	75.00		
Osteoporosis (n = 96)							
Optimal condition	4	2	50.00	2	50.00	0.429	0.934
Insufficiency	31	13	41.94	18	58.06		
Deficiency	33	13	39.39	20	60.61		
Significant deficiency	28	10	35.71	18	64.29		
Total (n = 287)							
Optimal condition	41	15	36.59	26	63.41	5.964	0.113
Insufficiency	92	44	47.83	48	52.17		
Deficiency	99	31	31.31	68	68.69		
Significant deficiency	55	19	34.55	36	65.45		

Note: n—total number; %—percentage representation; χ^2^—chi-square value; *p*—statistical significance; Total—total study population of postmenopausal women.

**Table 7:** Correlation analysis between total vitamin 25(OH)D concentration and selected parameters.

Group	Parameter	Parameter	r	*p*
CG	25(OH)D	BMI	0.113	0.376
		T-score LH neck	−0.1101	0.386
		T-score spine	−0.1625	0.200
		BMD LH neck	−0.111	0.384
		BMD spine	−0.164	0.196
OPE	25(OH)D	BMI	−0.047	0.606
		T-score LH neck	−0.045	0.622
		T-score spine	−0.182	0.042 *
		BMD LH neck	−0.050	0.579
		BMD spine	−0.177	0.048 *
OPO	25(OH)D	BMI	−0.085	0.409
		T-score LH neck	−0.072	0.486
		T-score spine	−0.180	0.079
		BMD LH neck	−0.068	0.508
		BMD spine	−0.139	0.176

Note: OPO—osteoporotic women group; OPE—osteopenic women group; CG—control group; BMI—body mass index; LH—left hip; r—correlation coefficient; *p*—statistical significance *.

## Data Availability

The original contributions presented in this study are included in the article/Supplementary Materials. Further inquiries can be directed to the corresponding authors.

## Supplementary Materials

The following supporting information can be downloaded at: https://www.mdpi.com/article/10.3390/genes16030337/s1, Table S1: The mean values of the monitored parameters in the individual genotypes of the *VDR* gene BsmI polymorphism in the OPO group; Table S2: Mean values of monitored parameters in individual genotypes of *VDR* gene BsmI polymorphism in the control group.

## Abbreviations

The following abbreviations are used in this manuscript:

BMD	Body mass density
BMI	Body mass index
VDR	Vitamin D receptor
CG	Control group (norm)
OPE	Osteopenic women group
OPO	Osteoporotic women group
PTH	Parathyroid hormone
LH	Left hip
25(OH)D total	Vitamin D total

## References

[1] Christakos S., Li S., De La Cruz J., Bikle D.D. (2019). New developments in our understanding of vitamin metabolism, action and treatment. Metab. Clin. Exp..

[2] Khammissa R.A.G., Fourie J., Motswaledi M.H., Ballyram R., Lemmer J., Feller L. (2018). The Biological Activities of Vitamin D and Its Receptor in Relation to Calcium and Bone Homeostasis, Cancer, Immune and Cardiovascular Systems, Skin Biology, and Oral Health. BioMed Res. Int..

[3] Holick M.F. (2007). Vitamin D deficiency. N. Engl. J. Med..

[4] Chen X., Shen L., Gao C., Weng R., Fan Y., Xu S., Zhang Z., Hu W. (2024). Vitamin D status and its associations with bone mineral density, bone turnover markers, and parathyroid hormone in Chinese postmenopausal women with osteopenia and osteoporosis. Front. Nutr..

[5] Al Anouti F., Taha Z., Shamim S., Khalaf K., Al Kaabi L., Alsafar H. (2019). An insight into the paradigms of osteoporosis: From genetics to biomechanics. Bone Rep..

[6] Brickley M.B., Mays S., George M., Prowse T.L. (2018). Analysis of patterning in the occurrence of skeletal lesions used as indicators of vitamin D deficiency in subadult and adult skeletal remains. Int. J. Paleopathol..

[7] Chowdhary R., Khan R.B., Masarkar N., Malik R., Goel S.K. (2022). An association of VDR gene polymorphism in hypovitaminosis D mediated secondary hyperparathyroidism in adolescent girls; a tertiary hospital study in central India. Steroids.

[8] Xiangpeng L., Zengli Z., Honghong Z., Hanmin Z., Jianlie Z. (2014). Application Guideline for Vitamin D and Bone Health in Adult Chinese (2014 Standard Edition) Vitamin D Working Group of Osteoporosis Committee of China Gerontological Society. Chin. J. Osteoporos..

[9] Cheng C.H., Chen L.R., Chen K.H. (2022). Osteoporosis Due to Hormone Imbalance: An Overview of the Effects of Estrogen Deficiency and Glucocorticoid Overuse on Bone Turnover. Int. J. Mol. Sci..

[10] Capatina C., Carsote M., Caragheorgheopol A., Poiana C., Berteanu M. (2014). Vitamin D deficiency in postmenopausal women—Biological correlates. Maedica.

[11] Melton L.J. (1995). How many women have osteoporosis now?. J. Bone Miner. Res..

[12] Banjabi A.A., Al-Ghafari A.B., Kumosani T.A., Kannan K., Fallatah S.M. (2020). Genetic influence of vitamin D receptor gene polymorphisms on osteoporosis risk. Int. J. Health Sci. (Qassim).

[13] Pike J.W., Meyer M.B., Benkusky N.A., Lee S.M., St John H., Carlson A., Onal M., Shamsuzzaman S. (2016). Genomic Determinants of Vitamin D-Regulated Gene Expression. Vitam. Horm..

[14] El-Hajj Fuleihan G., Bouillon R., Clarke B., Chakhtoura M., Cooper C., McClung M., Singh R.J. (2015). Serum 25-Hydroxyvitamin D Levels: Variability, Knowledge Gaps, and the Concept of a Desirable Range. J. Bone Miner. Res..

[15] Karohl C., Su S.Y., Kumari M., Tangpricha V., Veledar E., Vaccarino V., Raggi P. (2010). Heritability and seasonal variability of vitamin D concentrations in male twins. Am. J. Clin. Nutr..

[16] Al-Daghri N.M., Al-Attas O.S., Alkharfy K.M., Khan N., Mohammed A.K., Vinodson B., Ansari M.G., Alenad A., Alokail M.S. (2014). Association of VDR-gene variants with factors related to the metabolic syndrome, type 2 diabetes and vitamin D deficiency. Gene.

[17] Rahmadhani R., Zaharan N.L., Mohamed Z., Moy F.M., Jalaludin M.Y. (2017). The associations between VDR BsmI polymorphisms and risk of vitamin D deficiency, obesity and insulin resistance in adolescents residing in a tropical country. PLoS ONE.

[18] Zhao B., Zhang W., Du S., Zhou Z. (2016). Vitamin D receptor BsmI polymorphism and osteoporosis risk in post-menopausal women. Arch. Med. Sci..

[19] Marozik P.M., Tamulaitiene M., Rudenka E., Alekna V., Mosse I., Rudenka A., Samokhovec V., Kobets K. (2018). Association of Vitamin D Receptor Gene Variation with Osteoporosis Risk in Belarusian and Lithuanian Postmenopausal Women. Front. Endocrinol..

[20] Chandanwale R., Chandanwale K., Chandanwale R., Chandanwale A. (2024). Assessing the Correlation Between Anthropometric Measurements and Bone Densitometry as Indicators of Bone Health in Adult Women in the Community. Cureus.

[21] Reginster J.Y., Burlet N. (2006). Osteoporosis: A still increasing prevalence. Bone.

[22] Aghaei M., Afshan B., Reza H., Qorbani M., Dashti H.S., Safari R. (2013). Bone mineral density in Iranian patients: Effects of age, sex, and body mass index. Open J. Prev. Med..

[23] Baheiraei A., Pocock N.A., Eisman J.A., Nguyen N.D., Nguyen T.V. (2005). Bone mineral density, body mass index and cigarette smoking among Iranian women: Implications for prevention. BMC Musculoskelet. Disord..

[24] Steinschneider M., Hagag P., Rapoport M.J., Weiss M. (2003). Discordant effect of body mass index on bone mineral density and speed of sound. BMC Musculoskelet. Disord..

[25] Rico H., Revilla M., Villa L.F., Alvarez del Buergo M., Ruiz-Contreras D. (1994). Determinants of total-body and regional bone mineral content and density in postpubertal normal women. Metabolism.

[26] Henderson N.K., Price R.I., Cole J.H., Gutteridge D.H., Bhagat C.I. (1995). Bone density in young women is associated with body weight and muscle strength but not dietary intakes. J. Bone Miner. Res..

[27] Pluskiewicz W., Adamczyk P., Drozdzowska B. (2021). Height loss in postmenopausal women-do we need more for fracture risk assessment? Results from the GO Study. Osteoporos. Int..

[28] Fawzy T., Muttappallymyalil J., Sreedharan J., Ahmed A., Alshamsi A.A., Al Ali M.S., Al Balsooshi K.A. (2011). Association between Body Mass Index and Bone Mineral Density in Patients Referred for Dual-Energy X-Ray Absorptiometry Scan in Ajman, UAE. J. Osteoporos..

[29] Black D.M., Steinbuch M., Palermo L., Dargent-Molina P., Lindsay R., Hoseyni M.S., Johnell O. (2001). An assessment tool for predicting fracture risk in postmenopausal women. Osteoporos. Int..

[30] Montazerifar F., Karajibani M., Alamian S., Sandoughi M., Zakeri Z., Dashipour A.R. (2014). Age, Weight and Body Mass Index Effect on Bone Mineral Density in Postmenopausal Women. Health Scope.

[31] Kanis J.A., Cooper C., Rizzoli R., Reginster J.Y. (2019). Executive summary of European guidance for the diagnosis and management of osteoporosis in postmenopausal women. Aging Clin. Exp. Res..

[32] Nguyen N.D., Frost S.A., Center J.R., Eisman J.A., Nguyen T.V. (2007). Development of a nomogram for individualizing hip fracture risk in men and women. Osteoporos. Int..

[33] Nguyen N.D., Frost S.A., Center J.R., Eisman J.A., Nguyen T.V. (2008). Development of prognostic nomograms for individualizing 5-year and 10-year fracture risks. Osteoporos. Int..

[34] Adamczyk P., Werner A., Bach M., Żywiec J., Czekajło A., Grzeszczak W., Drozdzowska B., Pluskiewicz W. (2018). Risk factors for fractures identified in the algorithm developed in 5-year follow-up of postmenopausal women from RAC-OST-POL study. J. Clin. Densitom..

[35] Sadat-Ali M., Al Elq A.H., Al-Turki H.A., Al-Mulhim F.A., Al-Ali A.K. (2011). Influence of vitamin D levels on bone mineral density and osteoporosis. Ann. Saudi Med..

[36] Nair S., Bhadricha H., Patil A., Surve S., Joshi B., Balasinor N., Desai M. (2022). Association of OPG and RANKL gene polymorphisms with bone mineral density in Indian women. Gene.

[37] Gasperini B., Visconti V.V., Ciccacci C., Falvino A., Gasbarra E., Iundusi R., Brandi M.L., Botta A., Tarantino U. (2023). Role of the Vitamin D Receptor (VDR) in the Pathogenesis of Osteoporosis: A Genetic, Epigenetic and Molecular Pilot Study. Genes.

[38] Liu Y., Recker R., Deng H. (2003). Molecular Studies of Identification of Genes for Osteoporosis: The 2002 Update. J. Endocrinol..

[39] Uitterlinden A.G., Fang Y., Van Meurs J.B.J., Pols H.A.P., Van Leeuwen J.P.T.M. (2004). Genetics and Biology of Vitamin D Receptor Polymorphisms. Gene.

[40] Kamiński A., Bogacz A., Niezgoda-Nowak J.T., Podralska M., Górska A., Soczawa M., Czerny B. (2025). The *VDR* rs1544410 and rs11568820 Variants and the Risk of Osteoporosis in the Polish Population. Int. J. Mol. Sci..

[41] Kow M., Akam E., Singh P., Singh M., Cox N., Bhatti J.S., Tuck S.P., Francis R.M., Datta H., Mastana S. (2019). Vitamin D receptor (VDR) gene polymorphism and osteoporosis risk in White British men. Ann. Hum. Biol..

[42] Techapatiphandee M., Tammachote N., Tammachote R., Wongkularb A., Yanatatsaneejit P. (2018). *VDR* and *TNFSF11* polymorphisms are associated with osteoporosis in Thai patients. Biomed. Rep..

[43] Dehghan M., Pourahmad-Jaktaji R. (2016). The Effect of Some Polymorphisms in Vitamin D Receptor Gene in Menopausal Women with Osteoporosis. J. Clin. Diagn. Res..

[44] Moran J.M., Pedrera-Canal M., Rodriguez-Velasco F.J., Vera V., Lavado-Garcia J.M., Fernandez P., Pedrera-Zamorano J.D. (2015). Lack of association of vitamin D receptor BsmI gene polymorphism with bone mineral density in Spanish postmenopausal women. PeerJ.

[45] Seremak-Mrozikiewicz A., Drews K., Mrozikiewicz P.M., Bartkowiak-Wieczorek J., Marcinkowska M., Wawrzyniak A., Slomski R., Kalak R., Czerny B., Horst-Sikorska W. (2009). Correlation of vitamin D receptor gene (VDR) polymorphism with osteoporotic changes in Polish postmenopausal women. Neuro Endocrinol. Lett..

[46] Ahmad I., Jafar T., Mahdi F., Arshad M., Das S.K., Waliullah S., Mahdi A.A. (2018). Association of Vitamin D Receptor (FokI and BsmI) Gene Polymorphism with Bone Mineral Density and Their Effect on 25-Hydroxyvitamin D Level in North Indian Postmenopausal Women with Osteoporosis. Indian J. Clin. Biochem..

[47] Boroń D., Kamiński A., Kotrych D., Bogacz A., Uzar I., Mrozikiewicz P.M., Czerny B. (2015). Polymorphism of vitamin D3 receptor and its relation to mineral bone density in perimenopausal women. Osteoporos. Int..

[48] Gennari L., Becherini L., Masi L., Mansani R., Gonnelli S., Cepollaro C., Martini S., Montagnani A., Lentini G., Becorpi A.M. (1998). Vitamin D and estrogen receptor allelic variants in Italian postmenopausal women: Evidence of multiple gene contribution to bone mineral density. J. Clin. Endocrinol. Metab..

[49] Douroudis K., Tarassi K., Ioannidis G., Giannakopoulos F., Moutsatsou P., Thalassinos N., Papasteriades C. (2003). Association of vitamin D receptor gene polymorphisms with bone mineral density in postmenopausal women of Hellenic origin. Maturitas.

[50] Lisker R., López M.A., Jasqui S., Ponce De León Rosales S., Correa-Rotter R., Sánchez S., Mutchinick O.M. (2003). Association of vitamin D receptor polymorphisms with osteoporosis in mexican postmenopausal women. Hum. Biol..

[51] Shen H., Xie J., Lu H. (2014). Vitamin D receptor gene and risk of fracture in postmenopausal women: A meta-analysis. Climacteric: The journal of the International Menopause. Society.

[52] Fang Y., Rivadeneira F., van Meurs J.B., Pols H.A., Ioannidis J.P., Uitterlinden A.G. (2006). Vitamin D receptor gene BsmI and TaqI polymorphisms and fracture risk: A meta-analysis. Bone.

[53] Mondockova V., Kovacova V., Zemanova N., Babikova M., Martiniakova M., Galbavy D., Omelka R. (2023). Vitamin D Receptor Gene Polymorphisms Affect Osteoporosis-Related Traits and Response to Antiresorptive Therapy. Genes.

[54] Marozik P.M., Rudenka A., Kobets K., Rudenka E. (2021). Vitamin D Status, Bone Mineral Density, and VDR Gene Polymorphism in a Cohort of Belarusian Postmenopausal Women. Nutrients.

[55] Divanoglou N., Komninou D., Stea E.A., Argiriou A., Papatzikas G., Tsakalof A., Pazaitou-Panayiotou K., Georgakis M.K., Petridou E. (2021). Association of Vitamin D Receptor Gene Polymorphisms with Serum Vitamin D Levels in a Greek Rural Population (Velestino Study). Lifestyle Genom..

[56] Çakir M., Koç E.M., Soyöz M., Karahan Çöven H.İ., Aydogmus S., Sozmen K. (2023). The effects of VDR gene polymorphisms and Lifestyle features on vitamin D levels of postmenopausal women. Ankara Med. J..

