# Supplementary material for: Relationship Between Vitamin D Receptor Gene BsmI Polymorphism and 25-Hydroxyvitamin D Total Levels in Slovak Postmenopausal Women with Reduced Bone Mineral Density
Source: Genes (Basel). 2025 Mar 13;16(3):337. doi: 10.3390/genes16030337 (PMC11941902; doi:10.3390/genes16030337)
Supplement: Supplementary file 1 [file genes-16-00337-s001.zip › genes-3483887-supplementary.pdf]

Table S1 The mean values of the monitored parameters in the individual genotypes of the BsmI VDR gene polymorphism in the OPO group

Parameter	Genotype						*p*
	TT		TC		CC		
	n = 15		n = 43		n = 38		
	Mean	SD	Mean	SD	Mean	SD	
Age (years)	66.87	7.83	65.51	9.92	66.16	9.05	0.836
Onset of menopause (years)	47.27	9.00	47.91	4.98	49.00	3.44	0.744
Body weight (kg)	71.61	9.09	68.02	10.86	65.81	10.96	0.202
Body height (cm)	158.77	7.60	160.77	5.76	158.76	8.12	0.389
BMI (kg/m^2)	28.56	4.26	26.37	4.31	26.11	4.52	0.173
T-score LH neck	-1.83	0.53	-2.17	0.77	-2.21	1.00	0.310
T-score LH total	-0.77	1.11	-1.27	0.80	-1.14	0.82	0.162
Z-score LH neck	-0.36	0.51	-0.59	0.81	-0.62	0.99	0.232
Z-score LH total	0.49	1.22	0.13	0.89	0.11	0.88	0.379
BMD LH neck	0.65	0.06	0.61	0.09	0.60	0.11	0.320
BMD LH total	0.85	0.14	0.80	0.10	0.80	0.10	0.261
T-score spine	-2.11	0.57	-2.45	0.61	-2.55	0.78	0.103
Z-score spine	-0.23	0.94	-0.74	0.86	-0.77	1.02	0.151
BMD spine	0.82	0.06	0.78	0.07	0.77	0.09	0.084
PTH (pg/ml)	38.85	17.58	34.78	18.66	26.96	13.98	0.092
25(OH)D total (ng/ml)	14.76	6.07	16.57	9.62	18.46	15.72	0.809

n - number, SD - standard deviation, BMI - body mass index, kg – kilograms, cm – centimeters, LH - left hip, PTH - parathyroid hormone, 25(OH)D total - vitamin D total, p - statistical significance

Table S2 Mean values of monitored parameters in individual genotypes of BsmI VDR gene poly-morphism in control group

Parameter	Genotype						*p*
	TT		TC		CC		
	n = 8		n = 31		n = 26		
	Mean	SD	Mean	SD	Mean	SD	
Age (years)	54.00	6.07	58.74	8.69	59.92	10.60	0.293
Onset of menopause (years)	49.13	3.60	49.97	3.89	49.15	5.31	0.806
Body weight (kg)	80.63	13.13	85.29	17.36	78.72	12.69	0.262
Body height (cm)	160.75	4.36	162.40	7.22	162.38	6.50	0.808
BMI (kg/m^2)	31.26	5.31	32.53	7.28	30.04	5.74	0.358
T-score LH neck	-0.06	0.66	0.17	0.75	-0.16	0.71	0.169
T-score LH total	0.79	0.47	0.87	0.73	0.72	0.73	0.726
Z-score LH neck	0.91	0.77	1.39	0.92	1.17	0.86	0.250
Z- score LH total	1.48	0.66	1.81	0.85	1.74	0.81	0.595
BMD LH neck	0.84	0.07	0.87	0.08	0.83	0.08	0.180
BMD LH total	1.04	0.06	1.05	0.09	1.03	0.09	0.664
T-score spine	0.11	0.70	0.77	1.11	0.62	1.27	0.313
Z-score spine	1.15	0.99	2.17	1.38	2.10	1.38	0.122
BMD spine	1.06	0.08	1.13	0.12	1.11	0.14	0.338
PTH (pg/ml)	28.67	10.42	24.84	7.66	29.11	16.55	0.572
25(OH)D total (ng/ml)	20.14	10.39	16.57	8.30	22.05	11.02	0.164

n - number, SD - standard deviation, BMI - body mass index, kg – kilograms, cm – centimeters, LH - left hip, PTH - parathyroid hormone, 25(OH)D total - vitamin D total, p - statistical significance
